# Imidazolium-based ionic liquids used as additives in the nanolubrication of silicon surfaces

**DOI:** 10.3762/bjnano.8.197

**Published:** 2017-09-20

**Authors:** Patrícia M Amorim, Ana M Ferraria, Rogério Colaço, Luís C Branco, Benilde Saramago

**Affiliations:** 1 Centro de Química Estrutural, Instituto Superior Técnico, Universidade de Lisboa, Av. Rovisco Pais, 1049-001 Lisboa, Portugal; 2 LAQV-REQUIMTE, Departamento de Química, Faculdade de Ciências e Tecnologia, Universidade Nova de Lisboa, Campus da Caparica, 2829-516 Caparica, Portugal; 3 Centro de Química-Física Molecular and Institute of Nanoscience and Nanotechnology, Instituto Superior Técnico, Universidade de Lisboa, Av. Rovisco Pais, 1049-001 Lisboa, Portugal; 4 IDMEC-Instituto de Engenharia Mecânica, Departamento de Engenharia Mecânica, Instituto Superior Técnico, Universidade de Lisboa, Av. Rovisco Pais, 1049-001 Lisboa, Portugal

**Keywords:** additives, ionic liquids, lubricants, nanotribology, silicon

## Abstract

In recent years, with the development of micro/nanoelectromechanical systems (MEMS/NEMS), the demand for efficient lubricants of silicon surfaces intensified. Although the use of ionic liquids (ILs) as additives to base oils in the lubrication of steel/steel or other types of metal/ metal tribological pairs has been investigated, the number of studies involving Si is very low. In this work, we tested imidazolium-based ILs as additives to the base oil polyethylene glycol (PEG) to lubricate Si surfaces. The friction coefficients were measured in a nanotribometer. The viscosity of the PEG + IL mixtures as well as their contact angles on the Si surface were measured. The topography and chemical composition of the substrates surfaces were determined with atomic force microscopy (AFM) and X-ray photoelectron spectroscopy (XPS), respectively. Due to the hygroscopic properties of PEG, the first step was to assess the effect of the presence of water. Then, a series of ILs based on the cations 1-ethyl-3-methylimidazolium [EMIM], 1-butyl-3-methylimidazolium [BMIM], 1-ethyl-3-vinylimidazolium [EVIM], 1-(2-hydroxyethyl)-3-methylimidazolium [C_2_OHMIM] and 1-allyl-3-methylimidazolium [AMIM] combined with the anions dicyanamide [DCA], trifluoromethanesulfonate [TfO], and ethylsulfate [EtSO_4_] were added to dry PEG. All additives (2 wt %) led to a decrease in friction coefficient as well as an increase in viscosity (with the exception of [AMIM][TfO]) and improved the Si wettability. The additives based on the anion [EtSO_4_] exhibited the most promising tribological behavior, which was attributed to the strong interaction with the Si surface ensuring the formation of a stable surface layer, which hinders the contact between the sliding surfaces.

## Introduction

The use of ILs as neat lubricants was first proposed by Ye et al. in 2001 [1]. Since then, many investigations confirmed the good performance of ILs and their potential to substitute traditional lubricants in specific applications due to their peculiar properties. However, the price of ILs compared with that of commercial oils does not make them commercially competitive. Thus, the possibility of using ILs as additives to base lubricants rose as an attractive alternative from the economical point of view, and it has been pointed out in previous studies [2–3]. The first investigation reporting the use of ILs as additives is, to our knowledge, the work of Phillips et al. [4], who added imidazolium-based ILs to water for the lubrication of ceramics. Other investigators followed this idea and added imidazolium-based ILs to base oils to lubricate steel/aluminum contacts [5–7] and steel/steel contacts [8–13]. Jiménez and Bermúdez [7] tested two ILs with the short alkyl chain imidazolium cation [EMIM], and the anions tetrafluoroborate [BF_4_] and bis(trifluoromethylsulfonyl)imide [NTf_2_] mixed with propylene glycol dioleate to lubricate Al alloys. There was no impact on the friction coefficient but the wear rate was reduced significantly. The group of Liu [9–13] extensively investigated the role of ILs formed by imidazolium-based cations and the anions [BF_4_], hexafluorophosphate [PF_6_] and [NTf_2_] as additives to PEG and polyurea grease in the lubrication of steel/steel pairs, at room temperature and high temperatures. Recently, Pejaković et al. [14] tested several imidazolium sulfate ILs and found significant improvement of the tribological behavior of the same type of sliding pairs when 1-ethyl-3-methylimidazolium octylsulfate was added to the model lubricant fluid (glycerol). They attributed this to sulfur species in the tribofilm. Gusain et al. [15] synthesized bis-imidazolium ILs that proved to be efficient as additives to PEG in the lubrication of the same type of sliding pairs. Other ILs, namely those based on phosphonium [16–17] and ammonium [18–20] cations, have been tested as lubricant additives as described in recent reviews.

In most cases, the studies involved steel/steel or other types of metal/metal tribological pairs. However, the need for efficient lubrication of silicon surfaces rose with the development of micro/nanoelectromechanical systems (MEMS/NEMS) [21]. These miniaturized devices demand lubricants of high performance because the large surface-to-volume ratios may cause serious adhesion and friction problems, the so-called stiction. ILs stand up as promising lubricants for this type of systems, all the more so since ILs are conductive liquids, leading to the minimization of the contact resistance between sliding surfaces, which is required for various electrical applications. Several authors have successfully tested pure ILs as lubricants for Si surfaces [22–26]. However, to our knowledge, the only reports on the efficient behavior of ILs as oil additives to lubricate Si surfaces were recently published by the group of T. Atkin [27–28]. They studied the lubrication of silica surfaces at the macro- and the nanoscale with mixtures of trihexyl(tetradecyl)phosphonium bis(2,4,4-trimethylpentyl)phosphinate and apolar base oils, such as octane and hexadecane. Macroscale studies were done with a pin-on-disk tribometer under loads of 2 N and 10 N, while an atomic force microscope was used in the nanoscale investigation. Different lubrication regimes were observed at both scales: boundary lubrication at the nanoscale and mixed lubrication the macroscale. In the former case, lubricity was a function of the density of the adsorbed layer; in the latter one, the friction performance depended on the load: at low loads, the mixtures oil–IL were more efficient than the pure oil, while, at high loads, only concentrated oil–IL mixtures led to efficient lubrication.

**Figure 1:**
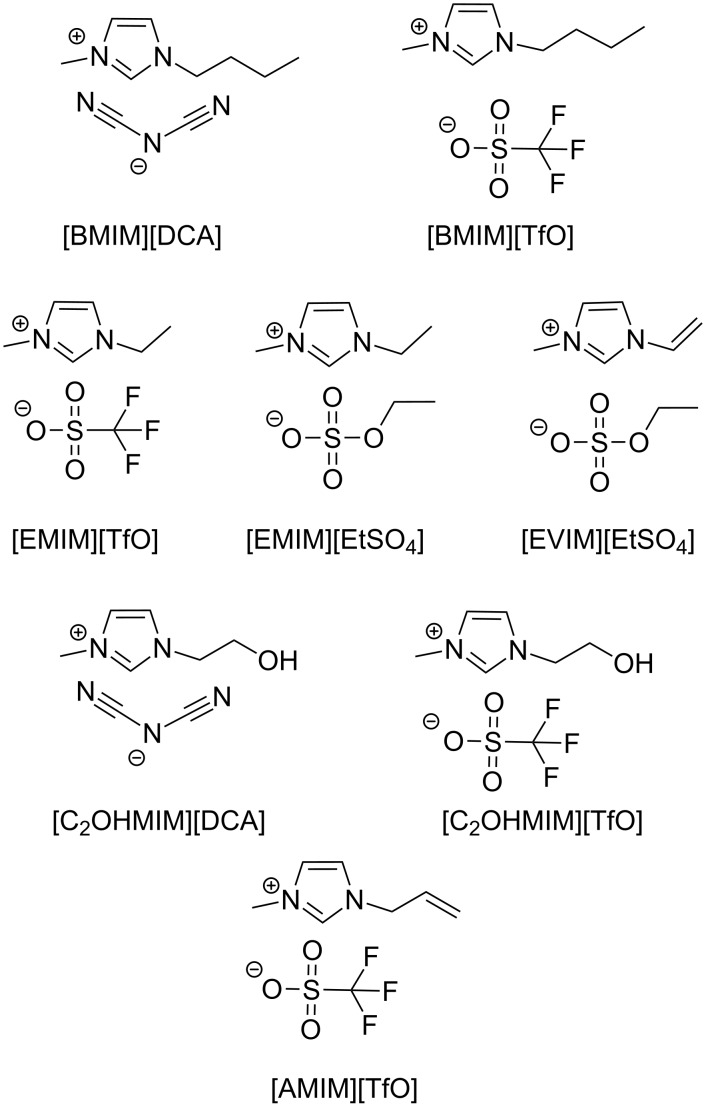
Structure of the studied ILs.

In this work, we went back to imidazolium-based ILs and tried to improve their tribological performance as additives through the introduction of adequate functional groups both in the cation and in the anion. First, we investigated the influence of the presence of water in the base oil (PEG) through the comparison of the behavior of dry and water-equilibrated mixtures. Then, a series of ILs based on the cations [EMIM], [BMIM], [EVIM], [C_2_OHMIM] and [AMIM] and the anions [DCA], [TfO], [EtSO_4_] were used as additives in PEG to lubricate Si surfaces using a nanotribometer. Figure 1 illustrates the structures of the studied ILs.

As far as we know, the lubricant properties of these pure ILs on silicon-based materials were never investigated, with the exception of [EMIM][EtSO_4_]. The reason should be the poor results obtained by other authors with similar ILs. Xie et al. [24] found a deficient lubrication performance of [EMIM][BF_4_], which was attributed to the small alkyl chain length on the imidazolium ring. Mo et al. [23] studied the lubricant properties of ILs based on the cation EMIM containing methyl, hydroxy, nitrile, and carboxyl groups deposited by dip-coating on Si surfaces. They found favorable friction reduction with [C_2_OHMIM][Cl] being the least efficient IL. In contrast, promising results were obtained with [EMIM][EtSO_4_], which were attributed to the presence of a stable tribofilm resulting from specific interaction between the [EtSO_4_] anion and the silicon surface [26,29].

The PEG + IL mixtures were characterized with respect to their viscosity and substrate wettability. The topography and chemical composition of the substrates surfaces were determined with atomic force microscopy (AFM) and X-ray photoelectron spectroscopy (XPS), respectively. Correlations between the obtained friction coefficients and the surface properties as well as the lubricants viscosity were attempted in order to understand the mechanism involved in the lubrication process.

## Results and Discussion

### Friction and wear under dry conditions

**Figure 2:**
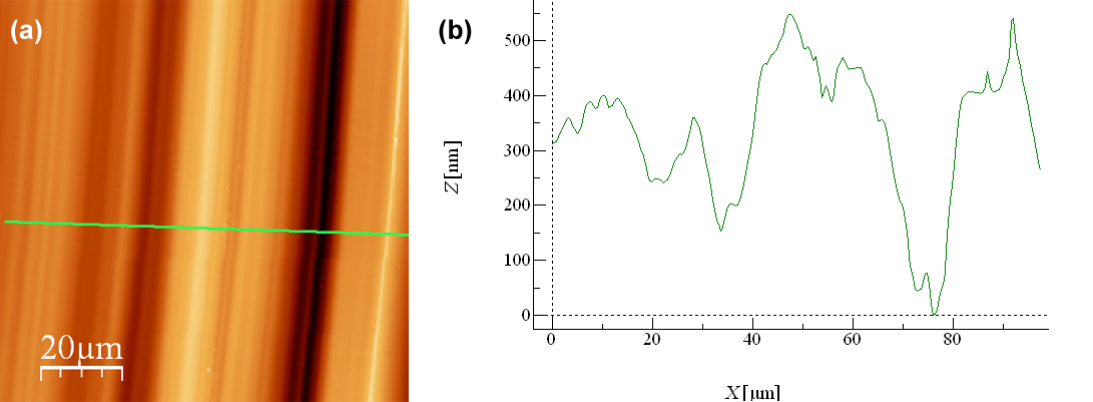
(a) AFM observation of the worn surface of Si submitted to nanotribological tests under dry conditions and (b) height profile of the worn region.

For comparison purposes, a set of nanotribological tests were done under dry conditions in a nitrogen stream using substrates and counter bodies similar to those used in the lubricated tests, the same normal load and scanning speed. The number of cycles used was 3100. In these tests it was observed that, after a very short running in period, the friction coefficient (CoF) stabilizes at a value of 0.7. Clear wear tracks could be observed, even with unaided eye, after the nanotribological tests under dry conditions. The AFM observation of these worn surfaces (Figure 2) reveals the existence of parallel grooves with peak-to-valley depths of approximately 500 nm, typical of severe abrasive conditions caused on the Si surface by the steel counter body.

### Effect of water in the lubricants

Most ILs and PEG itself are very hygroscopic liquids the water content of which may increase during the tribological applications under ambient conditions. Thus, it is important to know the influence of water on the tribological performance of pure PEG and its mixtures. Two types of PEG and PEG mixtures were tested: the liquids equilibrated with the atmosphere, here designated as humid, and the dry liquids, which were submitted to a vacuum drying process. Three ILs based on hydrophobic ([TfO]) and hydrophilic ([DCA]) anions were chosen as testing liquids to prepare the mixtures: [EMIM][TfO], [BMIM][TfO] and [BMIM][DCA]. The values of the viscosity, η, at 25 °C and the water content of humid and dry liquids (PEG and PEG + IL mixtures) are presented in Table 1.

**Table 1:** Viscosity, η, at 25 °C and water content of dry and humid liquids.

PEG + IL additive	humid liquids		dry liquids	
	η/mPa·s	water content/ppm	η/mPa·s	water content/ppm

PEG	36	4466	40	200–500
PEG + [EMIM][TfO]	48	3932	53	568
PEG + [BMIM][TfO]	47	3970	52	674
PEG + [BMIM][DCA]	52	4061	56	552

The presence of water decreases the viscosity of PEG and its mixtures by about 5 mPa·s. Addition of ILs increases the viscosity of humid and dry PEG in a similar fashion.

**Figure 3:**
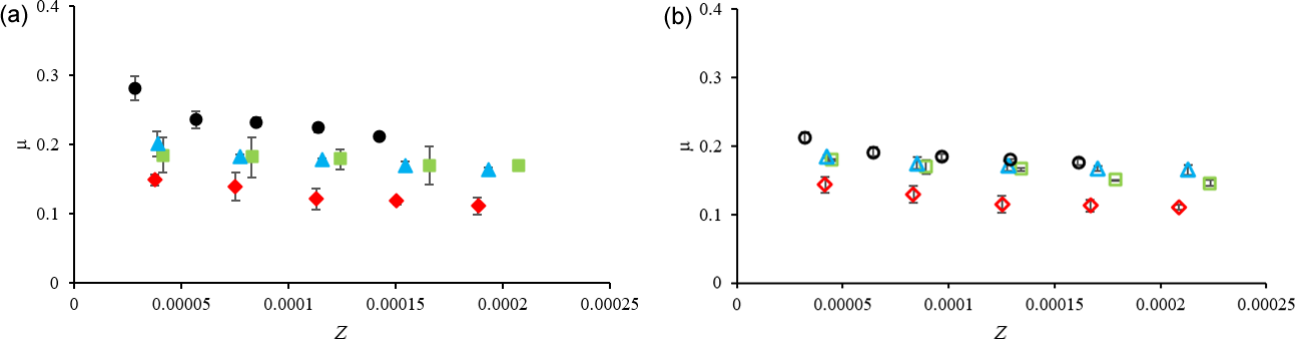
CoF as a function of the Sommerfeld parameter, *Z*, for (a) humid and (b) dry PEG (black circle) and PEG + [EMIM][TfO] (blue triangle), PEG + [BMIM][TfO] (red diamond), and PEG + [BMIM][DCA] (green square).

The first observation to retain from the nanotribological tests is the fact that, under the tested conditions, no wear was observed in any of the samples tested under lubricated conditions, either with PEG and with PEG+IL mixtures. In what concerns the CoF, the values are plotted in Figure 3. The values of CoF obtained with both humid and dry liquids are plotted as a function of the Sommerfeld parameter *Z* = η·*r*·*v* (Stribeck curves), where *F* is the applied force, *r* is the radius of the counterbody, and *v* is the velocity.

The main difference occurs between humid and dry PEG: The presence of water slightly increases the friction coefficient. Similar results were reported by the group of Spencer who found that water negatively affects the lubrication efficiency of ILs, at both the nanoscale [30] and the macroscale [31]. They used an extended surface force apparatus to study the influence of water on the structure of the nanoconfined films of ILs containing both hydrophilic ([EtSO_4_]) and hydrophobic (trifluorophosphate) anions. Water had different effects on both types of ILs: In hydrophilic ILs water hydrated the anions, while in hydrophobic ILs, it mainly hydrated the substrate surface. However, in both cases, ambient humidity was found to disturb the ion-pair coordination, which resulted in an increase in friction [31]. Tests carried out at macroscale showed the same effect when using hydrophobic imidazolium-based ILs to lubricate silica/silicon pairs at a small applied load (0.5 N). Under these conditions, low friction and no detectable wear in a nitrogen atmosphere was detected; in humid air, wear and friction increased, which was attributed to the disruption of lubricant film, leading to contact between the sliding surfaces [32]. Recently, the same group reported somewhat different results when studying the tribological behavior of silica/silicon pairs lubricated with [EMIM][EtSO_4_] with a pin-on-disk tribometer at high load (4.5 N) [32]. They studied the effect of ambient humidity and found a decrease in friction and wear of both counterparts when water was present in the IL which they attributed to the formation of a ductile layer of hydrated silica which resulted in the smoothing of the silica surface.

The increase in the viscosity of the dry liquids may also influence the tribological behavior, because in a mixed lubrication regime a more viscous lubricant would be more efficient in keeping the surfaces apart. Addition of ILs led to a decrease in friction, more significant for wet PEG, which should result from the formation of a boundary film as well as from the increase in the viscosity. In dry conditions an adsorbed layer of cations is expected; in wet conditions, adsorbed water may be present when the ILs are hydrophobic (TfO-based) or a layer of adsorbed cations and anions for hydrophilic ILs [31].

**Figure 4:**
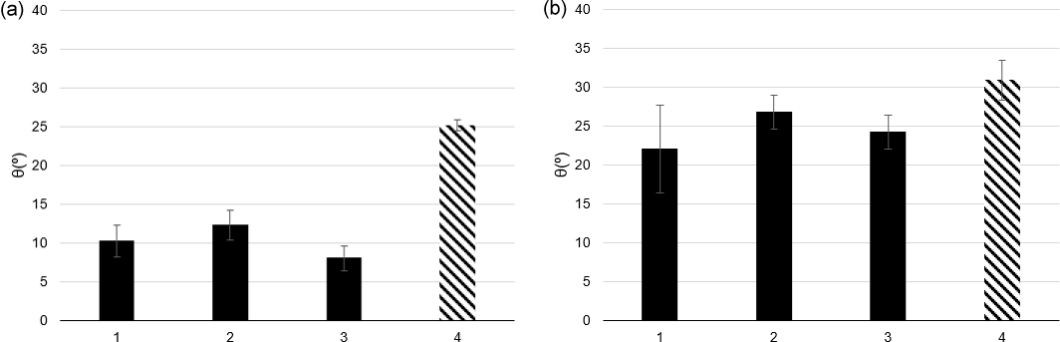
Contact angles of (a) humid and (b) dry PEG + [BMIM][DCA] (1), PEG + [BMIM][TfO] (2), PEG + [EMIM][TfO] (3), and PEG (4) on silicon substrates. The error bars correspond to standard deviations (*n* ≥ 7).

The contact angles of humid and dry PEG and their mixtures with ILs on the silicon substrates are presented in Figure 4. In both cases, addition of the ILs to PEG increased the wettability confirming the preferential interaction of the IL ions with the silicon surface. The contact angles of the humid liquids are lower than those of the dry liquids, which should result from the preferential adsorption of water molecules on the Si surface. The reduction of the contact angle in the presence of water is more significant for the PEG+IL mixtures than for pure PEG, which suggests that in the former case the water molecules being preferentially attached to the IL anions by H-bonds have a higher tendency to concentrate at the surface.

The correlation of the tribological performance with the substrate wettability is not a straightforward issue. In principle, for a boundary/mixed lubrication regime, a good wettability of the substrates should be important to keep a stable lubricating film. However, Borruto et al. [33] found that water lubrication in the mixed/hydrodynamic regime was most effective when pins and discs were made of materials of different wettability. In particular, when the counterbody was very hydrophilic and the disk very hydrophobic a stable layer of water remained between the sliding surfaces. In our case, the presence of water had a stronger effect on the CoF of pure PEG the wettability of which was the least affected. When comparing the different mixtures, both in dry or wet state, no correlation seems to exist between the substrate wettability and the tribological behavior, which is not unexpected considering the similarity of the corresponding contact angles.

### Effect of IL cation and anion

The ILs presented in Figure 1 were mixed with PEG (2 wt %) to prepare the mixtures used in the nanotribological tests. The choice of this concentration was based on preliminary tests using weight percentages of 1 wt %, 2 wt % and 5 wt %, which showed similar results for 2 wt % and 5 wt %. The characterization of the PEG + IL mixtures with respect to the viscosity, η, at 25 °C, water content and contact angle on the silicon surface is described in Table 2.

**Table 2:** Viscosity, η, at 25 °C, water content and contact angle of PEG + IL mixtures.

PEG + IL additive	η/mPa·s	water content/ppm	contact angle/°

PEG + [EMIM][TfO]	53	568	24 ± 2
PEG + [BMIM][TfO]	52	675	27 ± 2
PEG + [C_2_OHMIM][TfO]	55	466	15 ± 1
PEG + [AMIM][TfO]	40	406	17 ± 1
PEG + [BMIM][DCA]	56	552	22 ± 6
PEG + [C_2_OHMIM][DCA]	52	560	17 ± 2
PEG + [EMIM][EtSO_4_]	53	432	17 ± 2
PEG + [EVIM][EtSO_4_]	75	480	19 ± 3

The viscosity of the mixtures is considerably higher than that of PEG, except for PEG + [AMIM][TfO]. PEG + [EVIM][EtSO_4_] stands out as the most viscous mixture. All mixtures present lower contact angles than pure PEG (31°) on the silicon substrate, including those based on the hydrophobic [TfO] anion, especially when the cation contains hydroxy or allyl groups in the side chain.

**Figure 5:**
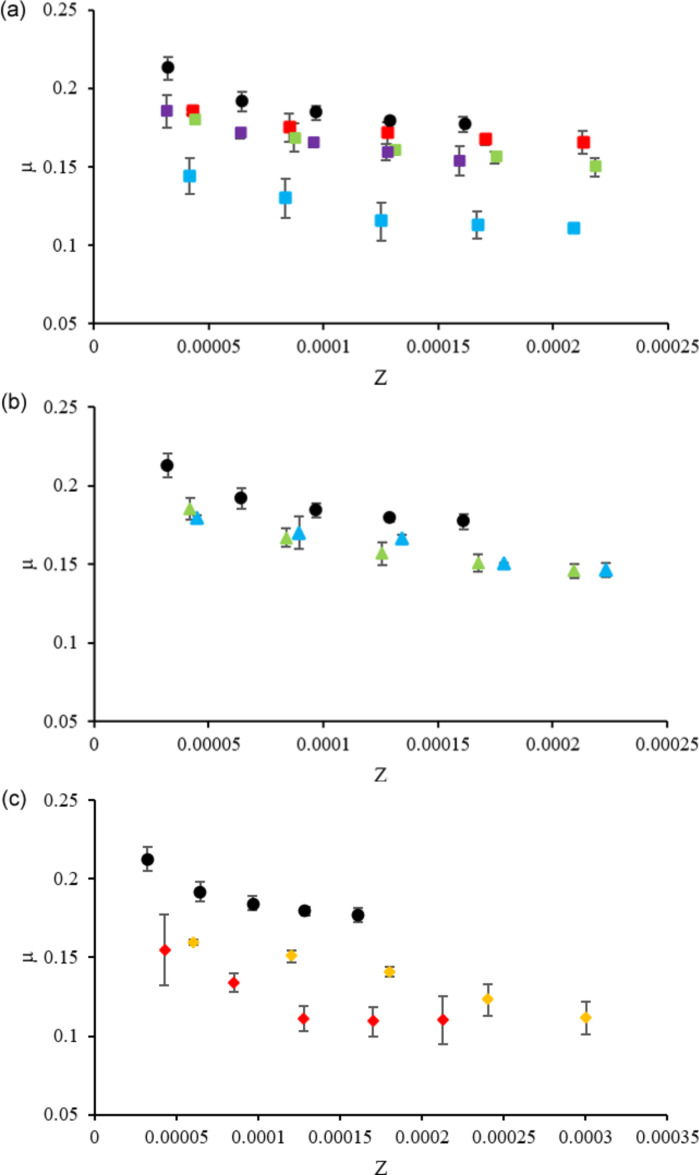
CoF vs Sommerfeld parameter, *Z*, for (a) TfO-based, (b) DCA-based and (c) EtSO_4_-based ILs mixed with PEG: PEG (black circles), PEG + [EMIM][TfO] (red squares), PEG + [BMIM][TfO] (blue squares), PEG + [C_2_OHMIM][TfO] (green squares), PEG + [AMIM][TfO] (purple squares), PEG + [BMIM][DCA] (blue triangles), PEG + [C_2_OHMIM][DCA] (green triangles), PEG + [EMIM][EtSO_4_] (red diamonds), PEG + [EVIM][EtSO_4_] (yellow diamonds).

In order to investigate the effect of the cation and the anion of the IL additive on the tribological performance of the PEG mixtures, we compared the CoFs obtained with PEG and with PEG+IL mixtures grouped according to the anion: ILs based on the hydrophobic [TfO] anion coupled with [EMIM], [BMIM], [AMIM] and [C_2_OHMIM]; ILs based the hydrophilic [DCA] anion coupled with [BMIM] and [C_2_OHMIM]; ILs based on the hydrophilic [EtSO_4_] anion coupled with [EMIM] and [EVIM]. The results are presented in Figure 5.

Analysis of Figure 5 indicates the presence of a mixed-lubrication regime, except for PEG + [EVIM][EtSO_4_] due to its high viscosity. Among the TfO-based additives (Figure 5a), the one with cation [EMIM] (shorter side chain) led to the worst results: It had almost no effect, in particular, at higher velocities. Addition to PEG of other cations with short side chains but functional groups (hydroxy or allyl), respectively, [C_2_OHMIM] and [AMIM], led to a slight decrease in friction. Cation [BMIM] with a longer side chain induced a significant decrease in friction, despite the fact that it presents the highest contact angle. Comparison of the DCA-based additives (Figure 5b) shows that all combinations ([BMIM] and [C_2_OHMIM]) led to similar small decreases in CoF. Finally, the EtSO_4_-based additives (Figure 5c) led to accentuated decreases in friction but the behavior of both cations was different. While [EVIM][EtSO_4_] led to a linear decrease of CoF with increasing *Z*, with [EMIM][EtSO_4_] a constant CoF was obtained for intermediate values of *Z*. This difference may be attributed to the higher viscosity of the former one which may contribute for the stability of the boundary layer at high sliding speeds.

**Figure 6:**
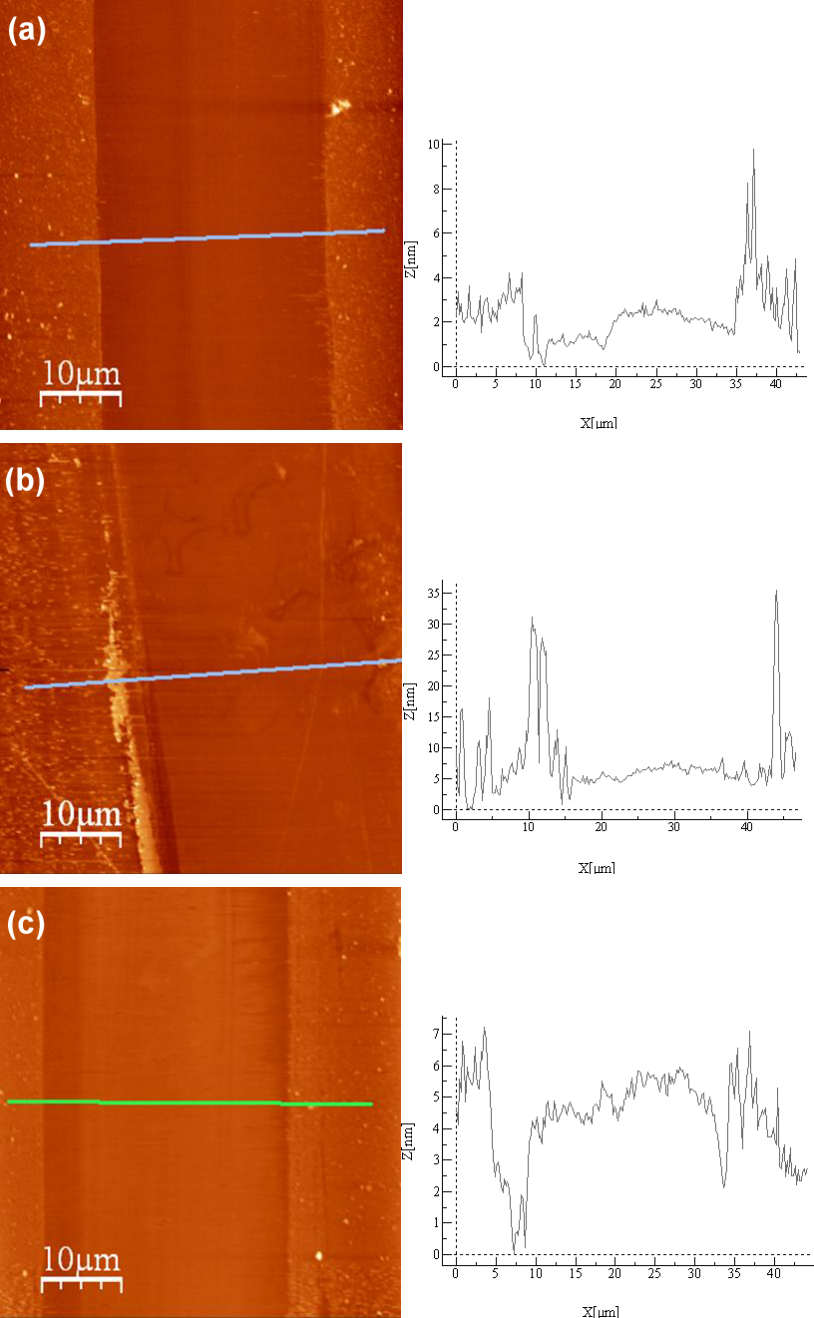
AFM images and height profiles of the sliding tracks in (a) dry PEG, (b) PEG + [EMIM][TfO] and (c) PEG + [EMIM][EtSO_4_].

To further understand the differences in behavior of the PEG/IL mixtures in the lubrication of the Si surface, AFM observation of some of the Si surfaces was done. After the nanotribological tests, tracks could be observed on the surfaces; however these tracks disappeared after washing the samples, demonstrating that no wear occurred. Figure 6 shows the AFM images of samples submitted to nanotribological tests (3100 cycles), before washing: Figure 6a shows the sample tested with pure PEG; Figure 6b shows the sample submitted to tribological tests performed with the mixture PEG + [EMIM][TfO], which presents a CoF similar to that of pure PEG (Figure 5a); Figure 6c shows the sample tested with the mixture PEG + [EMIM][EtSO_4_], which presents a lower CoF. Outside the sliding region an adsorbed layer with some agglomerates of the lubricant is present in all the samples. Moreover, AFM observation shows the following:

- Figure 6a**:** PEG adsorbs to the surface forming an adsorbed layer of approximately 3 nm of height. This layer is continuously removed by the sliding movement of the counter body. Nevertheless, PEG decreases the CoF and impairs the wear of the Si surface.
- Figure 6b**:** The surface of the samples submitted to tests with PEG + [EMIM][TfO] is similar to the surface of samples tested with PEG. Both the morphology of the layer and the depth of the sliding track (3–4 nm in average) are comparable to what can be observed in PEG samples, the main difference being the formation of some ridges at the periphery of the tracks, caused by the accumulation of the removed layer.
- Figure 6c**:** The adsorbed layer of PEG + [EMIM][EtSO_4_] is similar in morphology to the previous ones. However, in this case, the adsorbed layer is only partially removed in the periphery of the sliding tracks, indicating that the adhesion of PEG to the Si surface is enhanced by the presence of 2 wt % of [EMIM][EtSO_4_].

Therefore, the AFM results indicate that, although the adsorbed lubricant film is partially removed by the sliding action of the counterbody, in general, this removal is only partial and wear protection by the lubricants is ensured. The AFM observations also indicate that, when [EMIM][EtSO_4_] is added to PEG, the adsorbed layer is thicker which may explain the smaller CoF measured.

**Figure 7:**
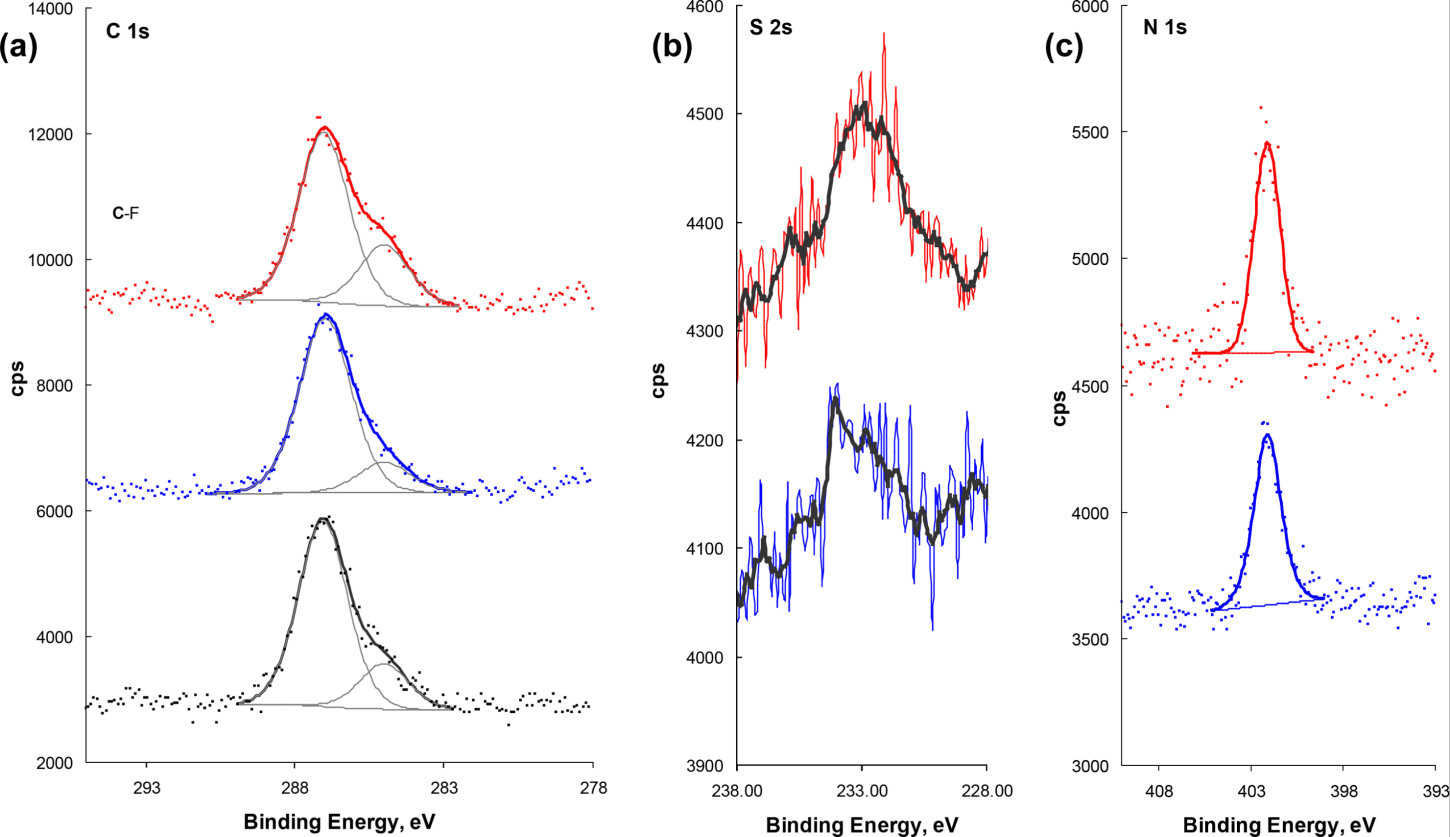
XPS regions of (a) C 1s, (b) S 2s, and (c) N 1s for, from bottom to top, in (a) Si/PEG, Si/PEG + [EMIM][EtSO_4_] and Si/PEG + [EMIM][TfO], and in (b) and (c) Si/PEG + [EMIM][EtSO_4_] and Si/PEG+[EMIM][TfO]. The solid black line in (b) represents the smoothed data.

XPS was used to analyze the Si surfaces after contacting with PEG and with two mixtures exhibiting the best and the worst nanotribological performance, respectively, PEG + [EMIM][EtSO_4_] and PEG + [EMIM][TfO] (see Figure 5). Adsorption of PEG to the Si surface was attested by the peak centered at about 287 eV assigned to carbon singly bound to oxygen (C–O) (Figure 7a), confirming the AFM observation. Another peak of C 1s, much smaller, is present at ca. 285 eV and may be assigned to hydrocarbon-like contaminations on the Si surface. In the case of the IL mixtures, no important differences exist among the C 1s signals: Two peaks may be fitted in the same positions, the intensity of the aliphatic carbon being lower in the PEG + [EMIM][EtSO_4_] sample. The most intense XPS sulfur peak is the S 2p (S 2p_3/2_ + S 2p_1/2_), which is superposed to the Si 2s plasmon loss. Comparison was, therefore, based on the S 2s peaks. In both samples, the signal to noise ratio is very poor (Figure 7b). Anyway, the smoothed data (full black lines) show different S 2s peaks in the sample treated with PEG + [EMIM][TfO] and with PEG + [EMIM][EtSO_4_]. In the former case, the peak centered at 232.9 eV, assigned to the sulfonate group in TfO, is broad but rather symmetrical. In the latter case, at least two peaks may be identified, one at higher energy (ca. 234 eV) assigned to sulfate, and the other one at lower energy similar to that of SO_3_ in TfO. This suggests that the anion EtSO_4_ is more affected by the presence of the substrate than the anion TfO. Finally, the spectra reveal a single N 1s peak centered at 402.1 eV, which is assigned to the nitrogen atoms with delocalized charge in the imidazolium ring of both [EMIM][EtSO_4_] and [EMIM][TfO] (Figure 7c). To further support this idea, quantitative atomic ratios were calculated and are given in Table 3.

**Table 3:** XPS atomic ratios for the Si samples coated with PEG, PEG + [EMIM][EtSO_4_] and PEG + [EMIM][TfO].

	PEG + [EMIM][EtSO_4_]	PEG + [EMIM][TfO]	PEG

C–O/Si	0.41 ± 0.01	0.43 ± 0.01	0.46 ± 0.01
N/C–O	0.13 ± 0.01	0.16 ± 0.01	—
S/C–O	0.042 ± 0.004	0.066 ± 0.007	—

The C–O/Si values given in Table 3 show that the amount of PEG on the Si surfaces exposed to the PEG + IL mixtures is slightly lower than that on the surface exposed to pure PEG, especially when the IL is [EMIM][EtSO_4_]. This result may be interpreted that either a smaller amount of polymer is adsorbed on the surface, or the same amount exists but PEG is more aggregated. The N/C–O and the S/C–O ratios suggest that less IL seems to exist in the sample coated with PEG + [EMIM][EtSO_4_] than in the sample coated with PEG + [EMIM][TfO]. Since the expected ratios should be similar in both cases, these results are compatible with stratification of the surface layers to some extent, with [EMIM][EtSO_4_] being closer to the Si surface than [EMIM][TfO]. This supports the interpretation given above for the large asymmetry of the S 2s peak in the former case. Stratification of ILs near solid surfaces, namely alumina and silica, was detected by several authors using atomic force microscopy imaging and MD simulations, even in the absence of long alkyl side chains [34–35]. However, stratification may lead to large distortions of the XPS atomic ratios when compared to the stoichiometric ones [36], thus no further quantitative analysis was performed.

Overall, these results seem to indicate a stronger interaction between [EMIM][EtSO_4_] and the surface compared with that of [EMIM][TfO], which leads to the greater stability of the adsorbed layer and the better tribological behavior of the additive [EMIM][EtSO_4_].

The efficiency of ILs based on the anion EtSO_4_ as pure lubricants was already reported in the literature [26,29]. However, to our knowledge, this is the first time that these ILs are successfully tested as additives to base lubricants. Strong adsorption of [EtSO_4_] on the surface oxide that covers the Si substrate should be responsible for this behavior. The S–O bond of this anion is known to interact with the silica surface to yield Si–O–S bonds, while the Si-OH groups were not found to give a significant contribution [37]. In this case, the positive role of the [EtSO_4_] anion seems to overlap the poor efficiency of the [EMIM] cation. Other authors [38–39] tested the frictional behavior of ILs composed of the cation [EMIM] and the sulfur-based anions, methylsulfate ([MeSO_4_]), *n*-buthylsulfate ([*n*-BuSO_4_]) and octylsulfate ([OcSO_4_]), as oil additives to lubricate steel surfaces and got different results. They found that friction did not improve with respect to that obtained with the base oil (glycerol), except under high shear conditions. Among the three ILs, the one based on [*n*-BuSO_4_] was the best, because it promoted a more complete covering of the substrate surface [38]. Later, they repeated the experiments keeping constant the molar fraction instead of the weight fraction of the additive and found that the mixtures with [EMIM][OcSO_4_] performed better because they formed thicker, more compact surface films. However, comparison with pure glycerol did not show a significant improvement [39]. All these results indicate the beneficial influence of the presence of IL additives depends strongly on the chemical nature of both ILs and the substrate. Enhanced interactions between the cation and or the anion and the hydrophilic silicon substrate, due to van der Waals forces (long side chains), hydrogen bonding (ethanol functional groups) and, more importantly, chemical bonding involving the hydrophilic sulfate and Si, promote the formation of an ordered surface layer, which helps to hinder the contact between the sliding surfaces.

## Conclusion

A series of imidazolium-based ILs was tested as additives to the base oil PEG in the lubrication of Si surfaces: [EMIM][TfO], [BMIM][TfO], [C_2_OHMIM][TfO], [AMIM][TfO], [BMIM][DCA], [C_2_OHMIM][DCA], [EMIM][EtSO_4_] and [EVIM][EtSO_4_]. The presence of water in PEG, equilibrated with the ambient atmosphere, worsened its tribological performance. However, contrary to what occurs in the tests under dry conditions, all the tested lubricants are efficient to impair wear under the tested conditions. A small percentage (2 wt %) of ILs was sufficient to induce a decrease in CoF, which was most significant in the case of those containing the anion [EtSO_4_]. XPS analysis of the silicon surfaces after exposure to PEG + IL mixtures containing the anions [EtSO_4_] and [TfO], respectively, leading to the best and the worst tribological behavior in what concerns CoF, confirmed the stronger interaction with [EtSO_4_]. The excellent results obtained with [EMIM][EtSO_4_] and [EVIM][EtSO_4_] encourage a deeper research on this type of additives with the objective of substituting the traditional oils in microelectronics lubrication.

## Experimental

### Materials

The following reagents were used: 1-methylimidazole (Alfa Aesar, 99%), 1-vinylimidazole (Sigma-Aldrich, 99%), allyl bromide (BHD, 99%), diethyl sulfate (Acros Organics, 99%), sodium dicyanamide (Aldrich, ≥97%). The solvents used for reaction media and/or purification processes are the following: dichloromethane (Carlo Erba Reagents, 99.9%), ethanol (LabChem), acetone (LabChem), acetonitrile (Carlo Erba Reagents, 99.8%), diethyl ether (LabChem, 99.9%). The reagent sodium trifluoromethanesulfonate, and the ILs [EMIM][TfO], [EMIM][EtSO_4_], [BMIM][TfO], [BMIM][DCA], [C_2_OHMIM][TfO], and [C_2_OHMIM][DCA] were kindly provided by Solchemar company (Portugal). The purity of these ILs is greater than 98%, according to the supplier. The commercial lubricant PEG 200 is from FLUKA (Sigma-Aldrich), ref: 81150. The substrates used for tribological tests and contact angle measurements are silicon wafers b100N with 0.5 mm of thickness and a roughness below 0.1 nm. Stainless steel AISI 316L spheres with 3 mm of diameter were used as counter bodies.

### Methods

#### Synthesis of ionic liquids

**[AMIM][Br]:** 1-Methylimidazole (14.0 mL, 176 mmol) was dissolved in 50 mL of acetone in a round-bottomed flask. This flask was submerged in ice and allyl bromide (15.2 mL, 211 mmol) was slowly added with stirring under an inert atmosphere. After the addition, the flask was fitted with a reflux condenser, removed from ice and kept at room temperature overnight. The next day, the mixture was kept at 40 °C for 5 h. In the end of the reaction the solvent was evaporated and the crude was washed with diethyl ether (5 × 5 mL under vigorous stirring) and then dried in vacuum at 85 °C for 3 days. The pure product was obtained as a viscous brown liquid (31 g, 87%).

^1^H NMR (DMSO-*d*_6_, 400 MHz) δ 3.90 (s, 3H), 4.94 (d, *J* = 8.00 Hz, 2H), 5.32 (m, 2H), 6.04 (m, 1H), 7.86 (d, *J* = 4.00 Hz, 2H), 9.42 ppm (s, 1H).

**[AMIM][TfO]:** [AMIM][Br] (8 g, 39.4 mmol) was dissolved in 50 mL of ethanol in an Erlenmeyer flask. Sodium triflate (7.8 g, 45.3 mmol) was also dissolved in 50 mL of ethanol, added to the previous solution and kept at room temperature under stirring for 24 h. In the end of the reaction the solvent was evaporated and the crude was dissolved in dichloromethane in which the sodium bromide precipitates. The solution was filtered and, after evaporation of dichloromethane, dried in vacuum at 85 °C for 3 days. The pure product was obtained as a brown viscous liquid (8.4g, 78%).

^1^H NMR (DMSO-*d*_6_, 400 MHz) δ 3.87 (s, 3H), 4.84 (d, *J* = 4.00 Hz, 2H), 5.33 (m, 2H), 6.04 (m, 1H), 7.70 (d, *J* = 4.00 Hz, 2H), 9.09 ppm (s, 1H); ^19^F NMR (DMSO-*d*_6_, 282 MHz) δ −77.99, −77.62 ppm; FTIR (KBr) 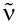: 518.86, 575.30, 642.20, 759.40, 848.18, 949.20, 995.75, 1033.18, 1166.81, 1261.74, 1426.68, 1573.08, 1643.73, 2361.52, 3115.17, 3156.87, 3511.69 cm^−1^; Anal. calcd for C_8_H_11_F_3_N_2_O_3_S·H_2_O: C, 33.10; H, 4.51; N, 9.65; found: C, 33.24; H, 4.08; N, 9.57.

**[EVIM][EtSO_4_]:** 1-Vinylimidazole (1.9 mL, 21.3 mmol) was dissolved in 20 mL of acetonitrile in a round-bottomed flask. Diethyl sulfate (3.6 mL, 27.6 mL) was slowly added at room temperature under stirring. After the addition, the flask was fitted with a reflux condenser for 48 h at 60 °C. In the end of the reaction the solvent was evaporated and the crude was washed with diethyl ether (5 × 5 mL under vigorous stirring) and then dried in vacuum at 85 °C for 3 days. The pure product was obtained as a viscous brown liquid (5.2 g, 99%).

^1^H NMR (DMSO-*d*_6_, 400 MHz) δ 1.10 (t, *J* = 12.00 Hz, 3H), 1.44 (t, *J* = 12.00 Hz, 3H), 3.79 (t, *J* = 12.00 Hz, 2H), 4.24 (m, 2H), 5.39 (m, 1H), 5.95 (m, 1H), 7.28 (m, 1H), 7.94 (s, 1H), 8.19 (s, 1H), 9.50 ppm (s, 1H); FTIR (KBr) 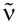: 585.28, 620.68, 781.43, 847.05, 919.84, 959.21, 1013.61, 1060.70, 1118.96, 1173.10, 1389.78, 1453.22, 1552.34, 1574.08, 1655.44, 2361.69, 2988.76, 3145.11, 3437.22 cm^−1^; Anal. calcd for C_8_H_14_N_2_O_4_S·(1.1H_2_O): C, 37.82; H, 6.43; N, 11.03; found: C, 37.85; H, 6.33; N, 10.53.

#### Characterization of IL mixtures

The water weight fraction of PEG and of the mixtures PEG + ILs, after being submitted to the vacuum drying process at 50 °C and 85 °C, respectively, was checked by Karl Fischer (coulometric) titration. The viscosity of PEG and its mixtures with the ILs was measured with a viscometer DV-II+Pro (Brookfield) at 25 °C. All measurements were done in triplicate. The temperature uncertainty was ±0.02 °C, while the precision of the dynamic viscosity measurements was ±0.5%.

The contact angle measurements on Si substrates were done with the sessile-drop method using the equipment described previously [40]. The Si substrates were submitted to a careful cleaning procedure: 2 × 15 min sonication in a detergent solution intercalated with 10 min sonication in water, followed by 3 × 10 min sonication in water, rinsing with distilled and deionized water, drying with nitrogen and finally drying for 2 h inside a vacuum oven at room temperature. The liquid drops were generated using a micrometric syringe from Gilmont inside an ambient chamber model 100-07-00 (Ramé-Hart, Succasunna, NJ, USA) at 20 °C. During the experiments, the chamber was continuously flushed with dry nitrogen, to minimize water absorption by the liquids. The images of drops were obtained with a video camera (jAi CV-A50, Barcelona, Spain) mounted on a microscope Wild M3Z (Leica Microsystems, Wetzlar, Germany) and analyzed by running the ADSA (Axisymmetric Drop Shape Analysis, Applied Surface Thermodynamics Research Associates, Toronto, Canada) software.

#### Nanotribological tests

The coefficients of friction (CoFs) were measured in a nanotribometer (CSM Instruments, Peseux, Switzerland) using PEG and PEG + ILs as lubricants. The Si substrates, previously cleaned as described above, were placed at the bottom of a liquid cell and a few drops of the lubricant were deposited on top to ensure complete coverage of the substrate surface. The amplitude of the reciprocal movement of the counter body (stainless steel sphere) on the Si surface was 0.5 mm and a force of 15 mN was applied. The sliding speed was varied between 0.4 and 2.0 cm·s^−1^, i.e., the nanotribological tests were carried out at low sliding speeds (low Sommerfeld numbers), corresponding to boundary or mixed-lubrication regimes. The number of cycles varied from 200 for the determination of CoF to 3100 for the observation of the substrates with AFM. Each test was repeated, at least, three times. The experiments were done at room temperature under a flow of dry nitrogen. The evolution of CoF with time was followed in all experiments and completely steady values were obtained after a few seconds meaning that the running in period was very short.

#### Microscopic observations

The surfaces of Si substrates after the nanotribological tests (3100 cycles) were observed with an optical microscope and then analyzed with an atomic force microscope (AFM) (NanoSurf Easyscan 2) using Si tips (*c* = 0.2 N·m^−1^, *f*_0_ = 25 kHz) at a constant contact force of 20 nN, in contact mode. The images were obtained through the WSxM 5.0 Develop 4.0 software.

#### XPS determinations

The Si substrates were immersed in PEG and PEG + IL for 40 min, which is the duration of the longer friction tests and then dried with a flux of Ar prior to XPS analysis. Spectra were acquired at TOA = 0° (relative to normal) with the Mg Kα X-ray source of a KRATOS XSAM800. Data treatment details were given in a previous work [41]. The sensitivity factors used for quantification analysis were: 0.318 for C 1s, 0.391 for S 2s, 0.736 for O 1s, 0.505 for N 1s and 0.371 for Si 2p.
